# Nanometre-scale pattern formation on the surface of a photochromic crystal by optical near-field induced photoisomerization

**DOI:** 10.1038/s41598-018-32862-9

**Published:** 2018-09-27

**Authors:** Ryo Nakagomi, Kazuharu Uchiyama, Hirotsugu Suzui, Eri Hatano, Kingo Uchida, Makoto Naruse, Hirokazu Hori

**Affiliations:** 1 University of Yamanashi, 4-3-11 Takeda, Kofu, Yamanashi 400-8511 Japan; 2 Ryukoku University, 1-5 Yokotani, Oe-cho, Seta, Otsu, Shiga 520-2194 Japan; 3 Network System Research Institute, National Institute of Information and Communications Technology, 4-2-1 Nukui-kita, Koganei, Tokyo 184-8795 Japan

## Abstract

We observed nanometre-scale optical near-field induced photoisomerization on the surface of a photochromic diarylethene crystal via molecular structural changes using an optical near-field assisted atomic force microscope. A nanometre-scale concavity was formed on the sample surface due to locally induced photoisomerization. By using this optical near-field induced local photoisomerization, we succeeded in generating a pattern of alphabet characters on the surface of the diarylethene crystal below the optical wavelength scale. Further, by exploiting the photochromism of the investigated material, erasure of the generated pattern was also confirmed, where the evolution of the pattern during erasure depended on the local spatial characteristics of the crystal. These experimental findings demonstrate the fundamental abilities of photochromic crystals in dynamic memorization in nanometre-scale light–matter interactions.

## Introduction

The unique physical attributes of light–matter interactions have recently been intensively studied with the objective of developing novel intelligent functionalities such as decision making^1,2^, solution searching^3^, and cognition^4,5^, among others. The reversible photoisomerization of photochromic materials is useful in functional optical systems. Among these materials, diarylethene is a promising vehicle because it is thermally stable and exhibits photochromic reactions in a crystalline state^6^. Moreover, upon near-field optical excitation of diarylethene crystals, the large momentum of the optical near-field^7–9^ causes local photoisomerization^10,11^, specifically, collective photoisomerization of the neighbouring molecules, leading to nanometre-scale mechanical distortion. The distortion suppresses the spreading of the photoisomerization to the surroundings, thereby maintaining the locality of the photoisomerization. In this article, we report on the optical near-field-induced, nanometre-scale photoisomerization observed on the surface of a diarylethene crystal as the local surface shape changed.

Before proceeding, we remark upon the concept behind our research. Ultimately, our objective is to realize novel optical functions on the nanometre scale using local distortions and photoisomerization in diarylethene crystals induced by near-field optical excitation. On the macroscopic scale, we previously succeeded in demonstrating decision-making functions using single photons^2^. By performing theoretical analysis, we showed that the decision-making architecture must include branching and multivaluedness^12^. The local photoisomerization triggered by near-field optical excitation of diarylethene crystals progresses in a complex manner along with branching and multivaluedness by spontaneous symmetry-breaking associated with anisotropic distortion on the nanometre scale, depending on the dynamic changes of the material structures, that is, environmental singularities. Therefore, nanometre-scale optical functionalities are likely to be achievable using diarylethene crystals. Furthermore, while photon information storage has been described in the literature as extremely difficult on all-optical processing^13^, nanoscale photochromism coupled with optical near-fields naturally enables memorization based on the intrinsic attributes of photochromic materials.

In our previous study, we observed a nanometre-scale complex photoisomerization pattern in a photochromic diarylethene crystal slab using a top-to-bottom double-probe scanning near-field optical microscope^14^. By inducing local near-field optical excitation on the front of the photochromic sample, we observed a subwavelength-scale two-dimensional pattern on the back of the sample that clearly differed from that formed by far-field light irradiation^14^. That is, the experimental results partially supported the feasibility of achieving the abovementioned nanometre-scale memorization using photochromism and near-field optical excitation. However, the excitation was performed only on a single spatial position, and experimental investigation of two-dimensional near-field addressing of local photochromism is necessary for spatial density and integration ability verification and for achieving a deeper understanding of optical near-field induced photoisomerization on the subwavelength scale.

In this study, we used diarylethene with a six-membered ring at the ethane moiety, which exhibits a photo-salient effect due to large molecular structural changes^15,16^, unlike conventional diarylethene, which has a five-membered ring at the centre. We consider large structural changes to enable the observation of local photoisomerization as local surface shape changes. In the experiment, we caused a local photoisomerization from closed-ring to open-ring isomers on the surface of a photochromic crystal. We observed the resulting 32-nm-diameter concavity using a light-assisted atomic force microscope (AFM). Furthermore, by two-dimensional addressing of the local near-field excitations, a nanometre-scale optical photoisomerization pattern of alphabet characters was formed on the crystal surface below the wavelength scale of the light. Moreover, the dynamic evolution was observed during erasure of the induced pattern, where each character exhibited a complex means of disappearance that depended on its shape. This study paves the way for achieving functional interplay involving optical near-fields and photoisomerization of diarylethene on the nanometre scale.

## Results and Discussion

**Figure 1:**
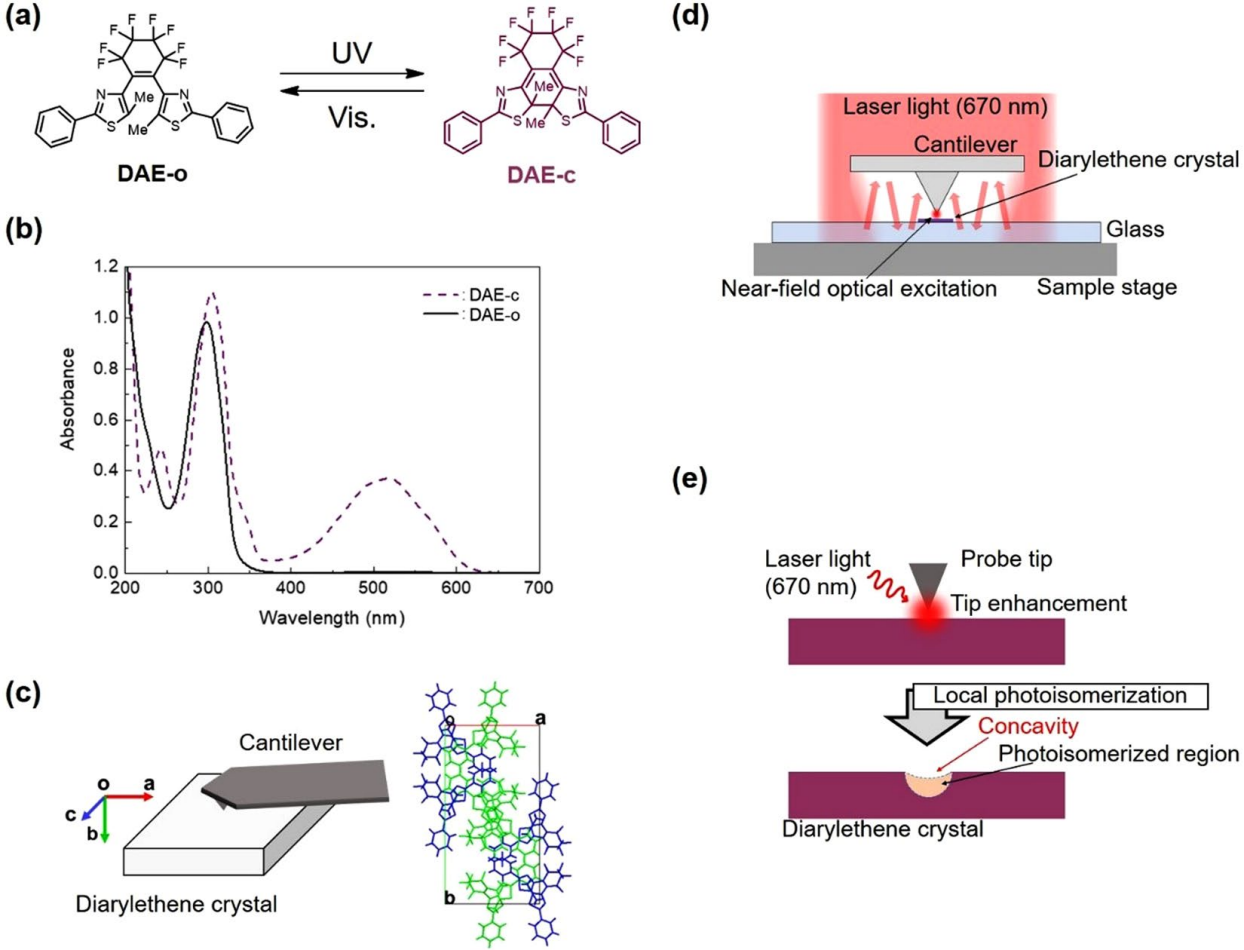
Experimental setup. (**a**) Molecular structures of DAE-o and DAE-c. (**b**) Absorption spectral changes of DAE-o in hexane (3.27 × 10^−5^ m). Absorption spectrum of DAE-o (solid line), DAE-c (broken line). (**c**) Observed crystal surface and molecular packing of two conformers in a unit cell. (**d**) Local excitation of the photochromic crystal by the optical near-field generated at the AFM probe tip. The laser light (wavelength: 670 nm) irradiated onto the upper surface of the cantilever was diffracted and reflected toward the tip and locally enhanced. (**e**) Concavity formation on the crystal surface by near-field optical excitation near the probe tip. The crystal surface is locally recessed, where the depth is proportional to the volume of the photoisomerized region.

The sample used in this study was a photochromic diarylethene single crystal, as shown in Fig. 1a, which has a six-membered ring at the ethane moiety^15,16^. The absorption spectrum in solution is shown in Fig. 1b. The correspondence between the crystal axis and observed surface is shown in Fig. 1c. Prior to the local optical excitation, the sample was reconfigured from the open-ring isomer of diarylethene (referred to as DAE-o) to the closed-ring isomer (DAE-c) by ultraviolet (UV) light irradiation. The local photoisomerization experiments described hereafter were focused on the transition from DAE-c (the coloured state) to DAE-o (the non-coloured state) on the crystal surface.

We used an optical-lever-type AFM (Bruker, Dimension Icon) for local optical addressing of the photochromic crystal. The cantilever was irradiated with laser light (wavelength: ~670 nm, intensity: ~1 mW), which was then locally enhanced at the probe tip to generate the optical near-field (see Fig. 1d). Hence, the photochromic molecules near the probe tip were excited by the optical near-field. Since photoisomerization involves a change in molecular structure, the crystal surface shape was slightly deformed in proportion to the photoisomerized region (see Fig. 1e). After keeping the probe tip close to a point on the crystal surface for from a few seconds to 1 min, we observed the surface by AFM and determined the local photoisomerization of the molecules near that point by observing the concavity generated on the surface.

**Figure 2:**
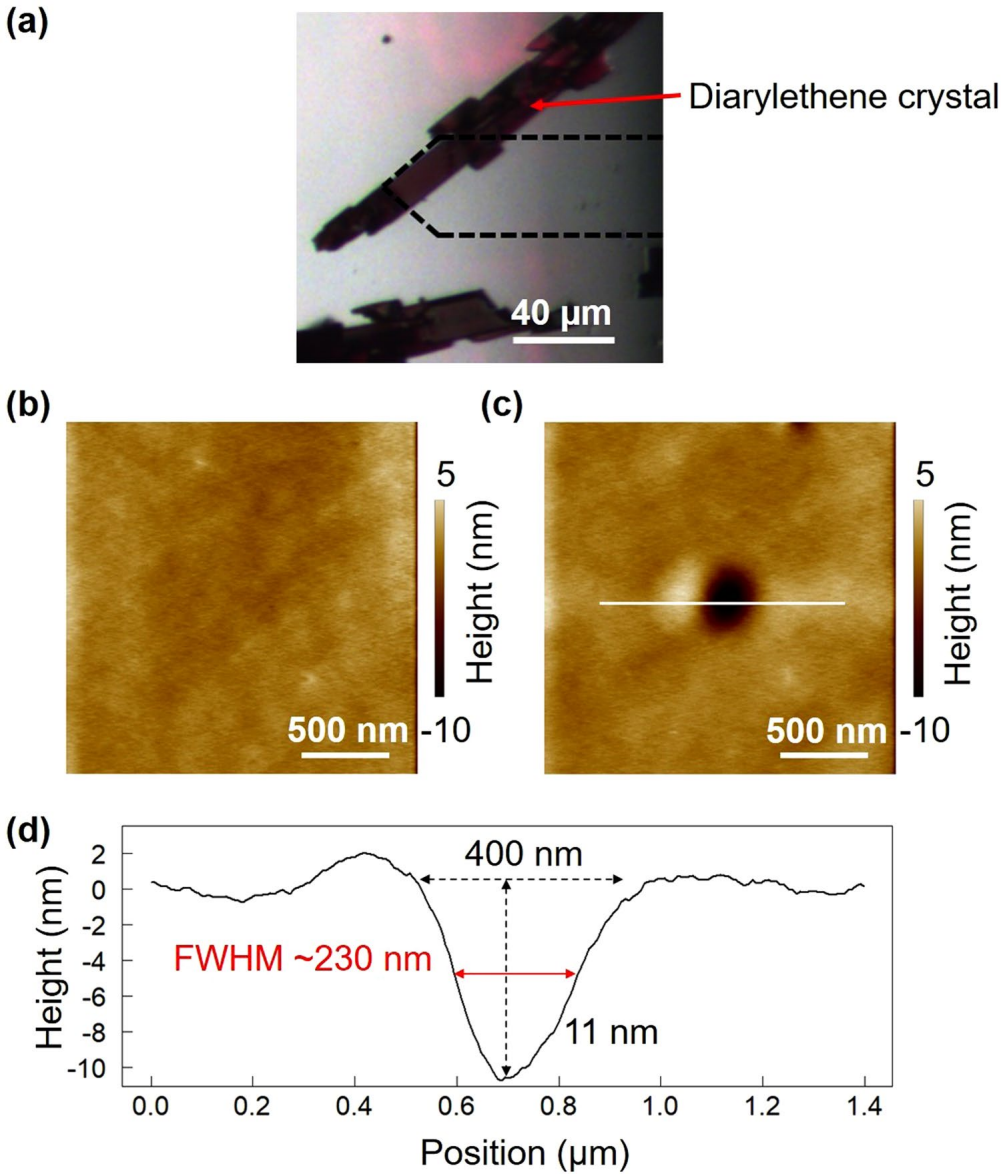
Effects of optical near-field excitation on a photochromic crystal. (**a**) Optical microscope image of the initial state of the crystal. The broken line shows the position of the cantilever. (**b**) AFM image of the crystal surface before local excitation. (**c**) AFM image after local excitation at the centre. (**d**) Line profile obtained along the white line in (**c**).

An optical microscope image of the crystal surface coloured by UV irradiation for 400 s with an UV light (Electro-Lite, Bondwand, wavelength: ~365 nm, intensity: ~10 mW/cm^2^) is shown in Fig. 2a, and an AFM image is presented in Fig. 2b. The AFM measurement conditions were as follows. The scanning area was 2 µm × 2 µm, the resolution was 256 px × 256 px (7.8 nm/px), and the scanning speed was 4 μm/s.

To induce local near-field optical excitation, the probe tip was placed near the centre of the region of interest for 60 s. We then observed the surface again to examine the effects of the local excitation. As shown in Fig. 2c, a concavity was generated at the probe tip location. A line profile of the surface structure around the concavity is provided in Fig. 2d. The concavity had an in-plane diameter of approximately 400 nm (full width at half-maximum, FWHM: ~230 nm) and a depth of 11 nm.

When the same experimental procedure was performed on an as-grown colourless sample, no such structural change was observed. That is, the concavity was generated by photoisomerization from DAE-c to DAE-o, due to the locally induced molecular structure change.

The shrinking of the observed surface due to the photoisomerization from the expanded coloured state (DAE-c) to the colourless state (DAE-o) is in agreement with the experimental observation of concavity formation. We consider the concavity depth to be proportional to the photoisomerization region depth. We speculated that the optical near-field at the tip excited the polarization mode in the nanometre-scale decoloured transparent part of the crystal under the tip as an extended optical near-field source. Hence, the part further beneath the surface was also photoisomerized.

In an attempt to achieve photoisomerization in a much smaller region, we reconfigured the initial colouring conditions. As described above, since the entire sample was initially coloured, decolourization by local excitation progressed to deep inside the crystal, resulting in the formation of a large concavity, which was larger than the tip of the near-field. We considered the initial colouring of only the crystal surface to be effective for the local photoisomerization from DAE-c to DAE-o, thereby enabling direct characterization of the locally induced near-field optical excitation.

**Figure 3:**
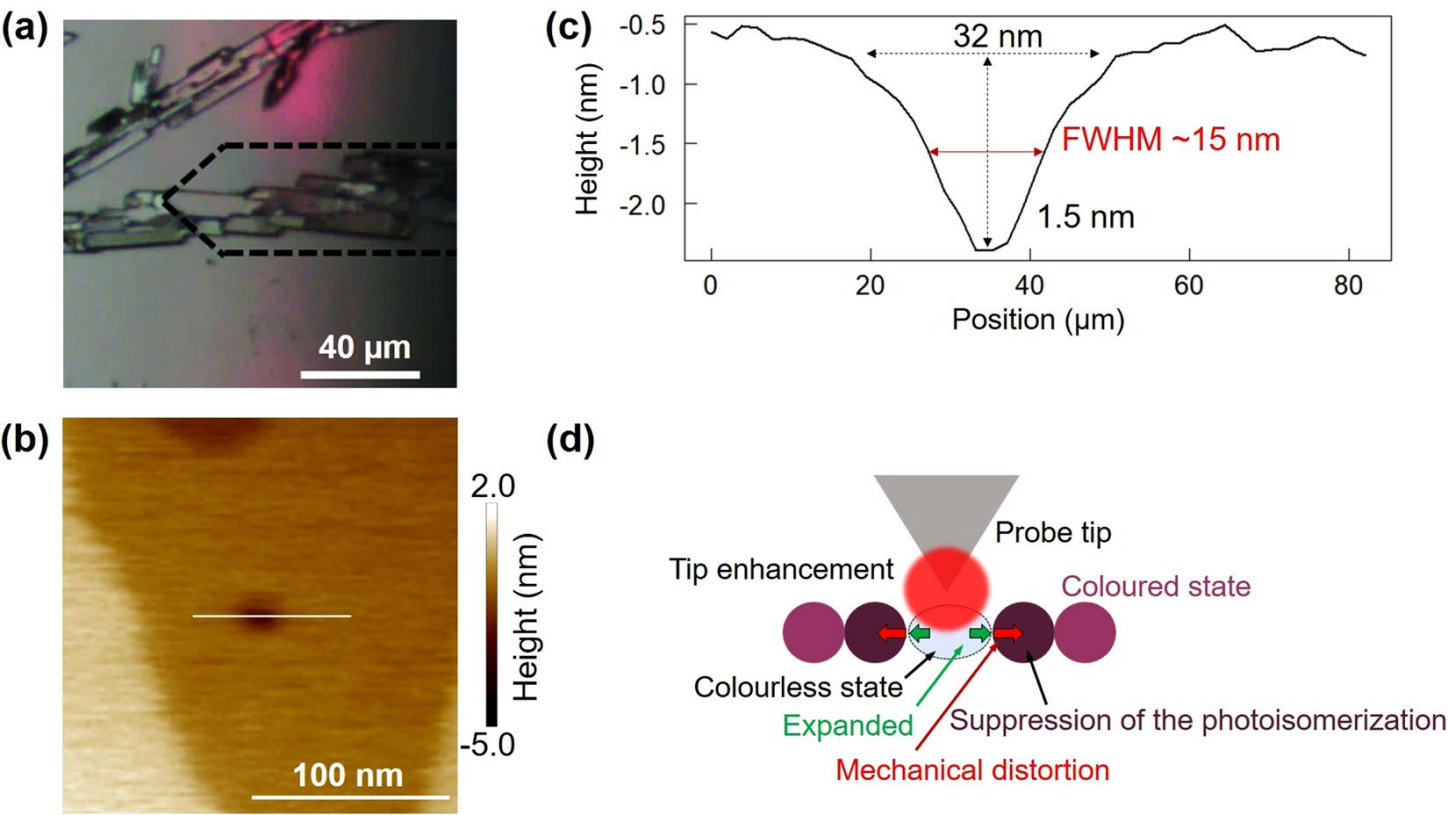
Local photoisomerization in a slightly coloured photochromic crystal. (**a**) Optical microscope image of the initial state of the crystal. (**b**) AFM image of the crystal surface after local excitation. (**c**) Line profile obtained along the white line in (**b**). (**d**) Mechanical distortion due to molecular structural change via local photoisomerization.

An optical microscope image of the crystal slightly coloured by weaker UV irradiation with a fluorescent lamp, corresponding to 1 s of irradiation with UV light, is shown in Fig. 3a. The probe tip was placed on the crystal surface for 30 s for near-field optical excitation. A small concavity is evident on the surface in the AFM image in Fig. 3b. In the AFM measurements, the scanning area was 500 nm × 500 nm, the scanning resolution was 256 px × 256 px (1.9 nm/px), and the scanning speed was 1 μm/s. The line profile around the concavity is presented in Fig. 3c. The concavity had an in-plane diameter of about 32 nm (FWHM: ~15 nm) and a depth of about 1.5 nm. This concavity was about 1/10 as large as that observed in the sample coloured for a longer time.

As schematically shown in Fig. 3d, the molecules photoisomerized collectively by near-field optical excitation expanded in the horizontal direction, and the mechanical distortion by expansion could then suppress the subsequent photoisomerization of the surrounding molecules, i.e. the extension of the decoloured region.

**Figure 4:**
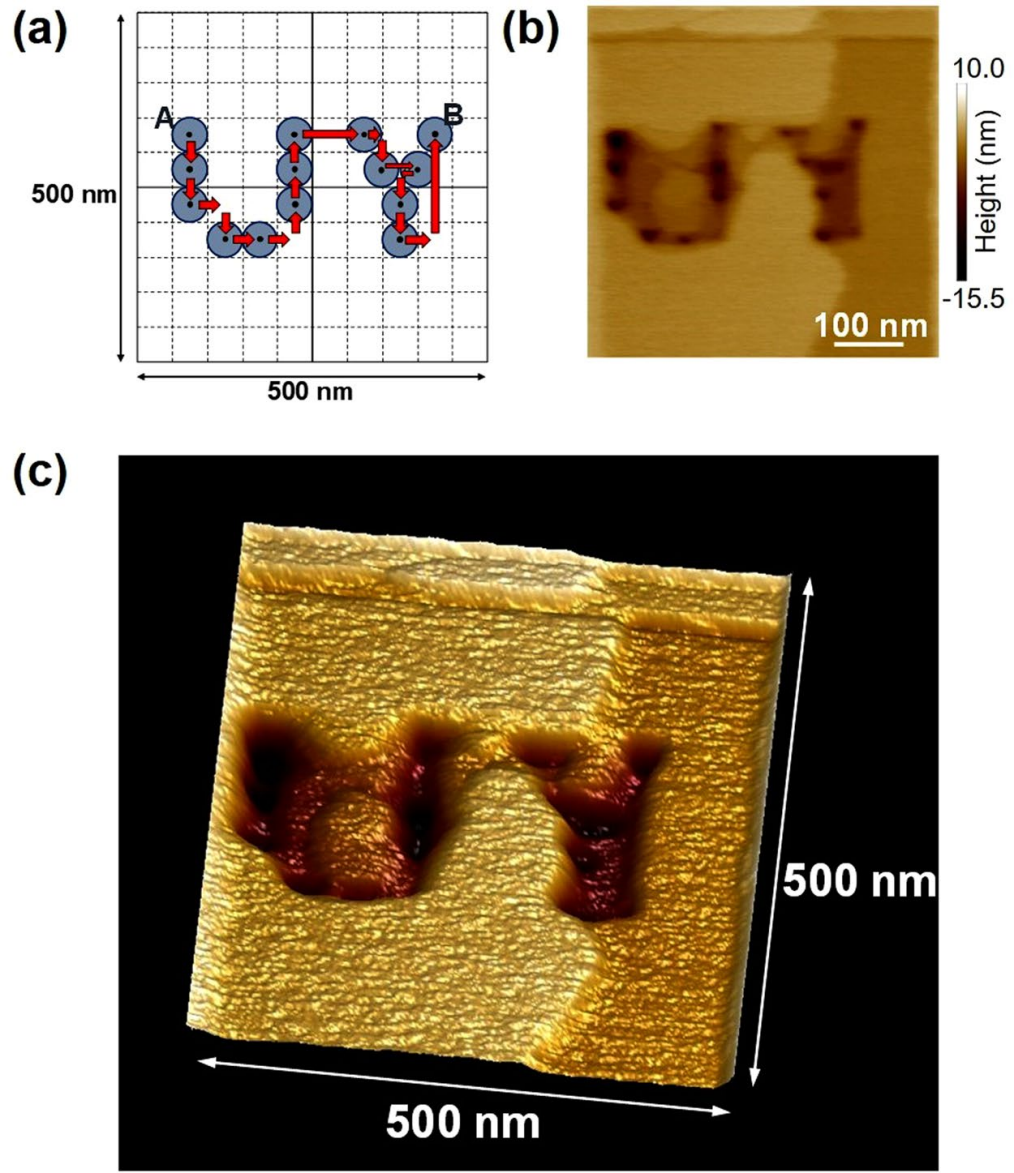
Photoisomerization pattern formation on a photochromic crystal surface by near-field optical excitation. (**a**) Trajectories of local optical excitations. (**b**) AFM image of the crystal surface with ‘UY’ drawn. (**c**) Three-dimensional image.

As demonstrated above, a local photoisomerization was achieved in the range of about 30 nm, which is far below the wavelength of the light. By exploiting such characteristics, we conducted fine-scale photoisomerization in the form of a two-dimensional pattern within the wavelength scale: we drew the alphabet characters ‘UY’ (the initials of the affiliation of the first author of this report) on the surface of the photochromic crystal. As shown in Fig. 4a, multiple excitation positions were configured with a pitch of 50 nm: the probe tip was placed for 64 s at the positions corresponding to the black dots to induce local near-field excitations. The location was sequentially changed from position A to position B as indicated by the arrows. The photochromic crystal was initially in a slightly coloured state, as in the aforementioned case in which the fine photoisomerization was achieved.

Figure 4b,c present AFM images depicting the characters ‘UY’ successfully drawn within the wavelength range of the light, demonstrating the high-density information memorization ability of the photochromic crystal. The AFM measurement conditions were as follows. The scanning area was 500 nm × 500 nm, the resolution of scanning was 256 px × 256 px (1.9 nm/px), and the scanning speed was 1 μm/s. To the best of our knowledge, this is the smallest pattern formed on any photochromic material^17,18^. By examining the details, linear grooves can be discerned in the lower right corner of ‘U’ and on the right side of ‘Y’. These correspond to the probe tip trajectories shown in Fig. 4a. The probe speed was about 2 µm/s, so the excitation time corresponding to a length of 50 nm was less than 25 ms. Thus, optical near-field photoisomerization could be performed in 1 s or less in several molecular layers.

We drew the pattern ‘UY’ by decolourizing part of the coloured surface by near-field optical. We confirmed disappearance of ‘UY’ by complete decolourization of the entire surface with near-field optical excitation of the continuous AFM observation.

**Figure 5:**
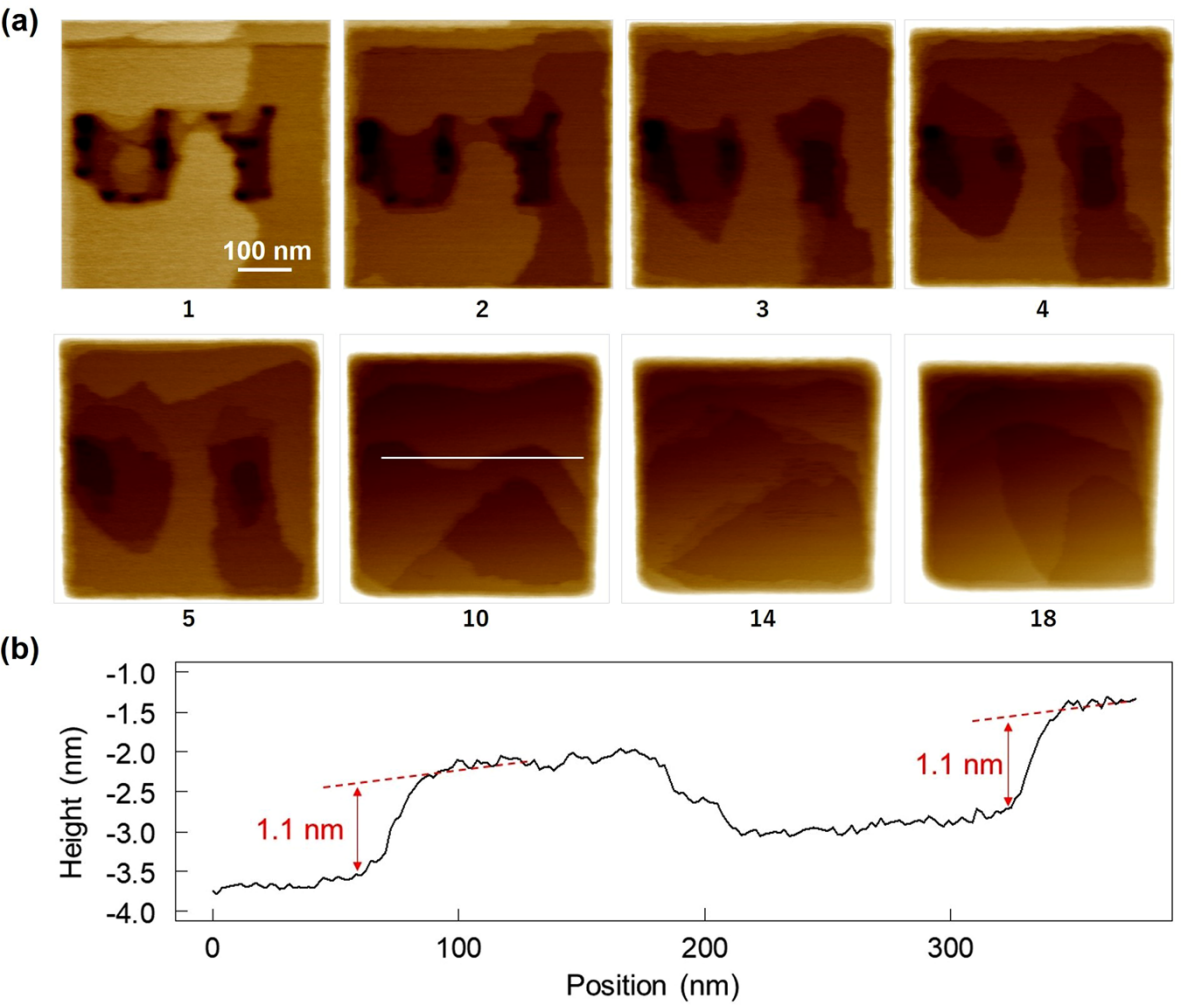
Dynamic erasing of nanometre-scale patterns formed on a photochromic crystal. (**a**) Sequential AFM images of the surface where the ‘UY’ pattern was formed. The number under each image is the number of that frame in the series. (**b**) Line profile obtained along the white line in the 10th frame.

Figure 5a presents eight of the obtained AFM images, where the subscripts indicate the frame numbers. Each AFM measurement took 260 s. Characteristic shapes remain until the third frame, and the alphabet characters become almost indistinguishable at the fourth frame. At the 10th frame, even the drawing areas of the characters cannot be identified and the surface appears to have been reconstructed as an unspecific surface composed of terrace structures of 100 nm or more. In these manners, the original pattern disappeared according to a unique evolution.

This series of images obtained while erasing ‘UY’ illustrates the disappearance of singularity and control of non-triviality. For example, the surface pattern in the fourth frame is thought to contain various information, including several characters such as ‘UY’ and ‘HI’. Thus, it is possible to control the degrees of singularity and non-triviality that give meanings to things. Meanwhile, the evolving pattern indicates the time elapsed since the erasure began. These local photoisomerization features contribute to the realization of dynamic history memorization with advanced information processing ability.

Figure 5b shows a line profile obtained along the crystal surface in the 10th frame: the patterns on the crystal surface are composed of molecular-scale flat terraces with steps of about 1 nm. That is, the process of erasing the character pattern ‘UY’ consisted of a sequence of generating and displacing such discrete molecular-scale steps. Such a 1-nm-scale step structure was also previously reported when comparing the surface morphologies of coloured and decoloured states in a crystal composed of another diarylethene material; this structure is considered to be related to the fundamental unit of collective photoisomerization of molecules in crystals^19^.

With optical near-field excitation, photoisomerization is locally induced along with local mechanical distortions. Moreover, the 1-nm-scale material-originated characters were inherent. We consider the versatile evolution of the observed patterns (as in Fig. 5a) to have been generated at the subwavelength scale due to the combination of these optical and material characteristics.

In summary, we observed nanometre-scale local photoisomerization from DAE-c to DAE-o on the surface of a photochromic single crystal by employing a light-assisted AFM. A concavity with a FWHM of 15 nm and a depth of 1.5 nm was generated via a locally induced optical near-field. In addition, we succeeded in drawing alphabet characters on a scale less than that of the wavelength of the light by sequential optical near-field excitations on the photochromic crystal surface. Furthermore, a unique evolution process was observed during erasure of the drawn pattern by optical near-field excitation, where 1-nm-scale terrace structures were clearly observed in the photochromic material. These findings demonstrate that photochromic diarylethene crystals provide a platform for realizing dynamic memorization that reflects past input/output relationships expected for artificially constructed intelligent nanoscale devices. In the future, we will examine further experimental considerations, including, for instance, local switching of decoloured molecules into the coloured state by UV irradiation, instead of decolouring all of the surrounding molecules as demonstrated in the present study, to enable realization of the full benefits of the reversibility of locally induced near-field photochromism.

## Methods

### Sample

The colourless open-ring isomer of diarylethene (DAE-o) underdoes cyclization to the red-purple coloured closed-ring isomer (DAE-c) upon ultraviolet (UV) irradiation (as shown in Fig. 1a). DAE-c reverts to DAE-o upon visible light irradiation. The diarylethene was prepared according to the methods described in previous papers^15,16^. Several types of DAE-o crystals were generated on glass by sublimation. (The details are presented in Supplementary Information). Observations were performed on the surfaces of rod-shaped crystals (see Supplementary Fig. S1) grown along the glass surface (as shown in Fig. 1c). The largest plane of a DAE-o crystal is the (010) surface^15,16^. Upon UV irradiation of the surface, the crystal expands along the *a*- and *b*-axes, shrinks along the *c*-axis, and undergoes photochromic colouration from DAE-o to DAE-c.

### Atomic force microscope (AFM)

The AFM measurements were performed in tapping mode (drive frequency: ~287 kHz) with a probe composed of Sb-doped Si (Bruker, RTESPA-300, tip radius: ~8 nm, spring constant of the cantilever: ~40 N/m, resistivity: ~0.01–0.025 Ωcm).

## Electronic supplementary material

Supplementary Information

## Data Availability

The datasets generated during the current study are available from the corresponding author on reasonable request.
