# Author Correction: Nanometre-scale pattern formation on the surface of a photochromic crystal by optical near-field induced photoisomerization

**DOI:** 10.1038/s41598-018-35959-3

**Published:** 2018-11-27

**Authors:** Ryo Nakagomi, Kazuharu Uchiyama, Hirotsugu Suzui, Eri Hatano, Kingo Uchida, Makoto Naruse, Hirokazu Hori

**Affiliations:** 1 University of Yamanashi, 4-3-11 Takeda, Kofu, Yamanashi, 400-8511 Japan; 2 Ryukoku University, 1-5 Yokotani, Oe-cho, Seta, Otsu, Shiga, 520-2194 Japan; 3 Network System Research Institute, National Institute of Information and Communications Technology, 4-2-1 Nukui-kita, Koganei, Tokyo, 184-8795 Japan

Correction to: *Scientific Reports* 10.1038/s41598-018-32862-9, published online 27 September 2018

In Figure 3C, the horizontal axis is incorrectly labelled as ‘Position (µm)’. This should read ‘Position (nm)’. The correct Figure 3C appears below as Figure 1.

**Figure 1:**
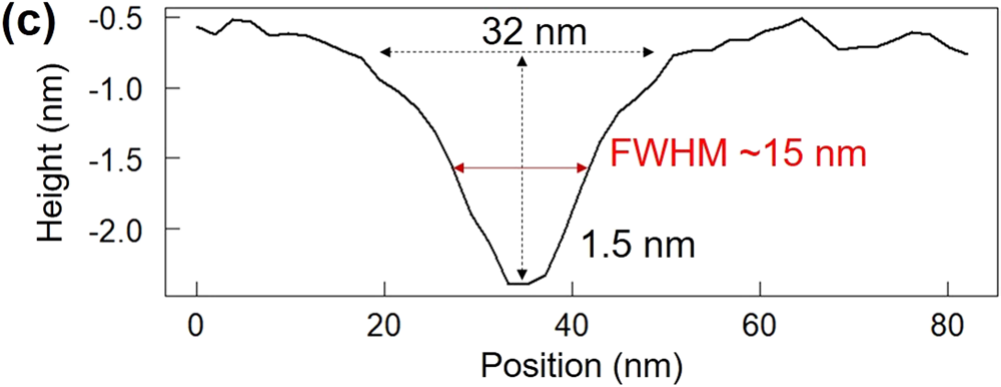
Local photoisomerization in a slightly coloured photochromic crystal. (**c**) Line profile obtained along the white line in (**b**).

