# Supplementary material for: Nanometre-scale pattern formation on the surface of a photochromic crystal by optical near-field induced photoisomerization
Source: Sci Rep. 2018 Sep 27;8:14468. doi: 10.1038/s41598-018-32862-9 (PMC6160423; doi:10.1038/s41598-018-32862-9)
Supplement: Supplementary file 1 — Supplementary Information [file 41598_2018_32862_MOESM1_ESM.docx]

**Supplementary Information**

**Article in *Scientific Reports***

**Nanometre-scale pattern formation on the surface of a photochromic crystal by optical near-field induced photoisomerization**

Ryo Nakagomi, Kazuharu Uchiyama, Hirotsugu Suzui, Eri Hatano, Kingo Uchida, Makoto Naruse, and Hirokazu Hori

In this supplementary information, we present experimental details.

1. **Preparation of crystals of DAE**

The formation process of the crystals of DAE-o by sublimation is schematically illustrated in Fig. S1. In a 26 × 26 × 1.2 mm space between slide glasses, 1.0 mg of DAE-o was scattered. Then, the glasses were heated on a hotplate for 90 min at 138 °C, which is 20 °C lower than the melting point of DAE-o (158 °C). Four types of crystals of DAE-o were generated on the glass plate on the top; Specifically, (A) rod-shaped crystals grown along the glass surface, (B) hollow crystals grown along the glass surface, (C) hollow crystals grown standing on the surface, and (D) platelet crystals standing on the surface. Crystals (B), (C), and (D) were grown at the same growing point. In this study, crystal (A) was used.

1. **Molecular-level specifications of the photochromic crystal used in the study**

Table S1 summarizes the molecular-level characteristics of the photochromic crystal used in the present study obtained by an X-ray diffractometer (Bruker AXS, D8 QUEST). Correspondingly, a three-dimensional schematic diagram of the molecular packing is illustrated in Fig. S2.

1. **Absorption spectrum of the DAE in crystalline state**

The absorption spectrum of the DAE in its crystalline state is shown in Fig. S3.

**Reference**

1. Hatano, E. *et al.* Photosalient effect of a diarylethene with a perfluorocyclohexene ring. *Chem. Eur. J.* **22,** 12680–12683 (2016).
2. Hatano, E. *et al.* Photosalient phenomena that mimic impatiens are observed in hollow crystals of diarylethene with a perfluorocyclohexene ring. *Angew. Chem. Int. Ed.* **56,** 12576–12580 (2017).

**
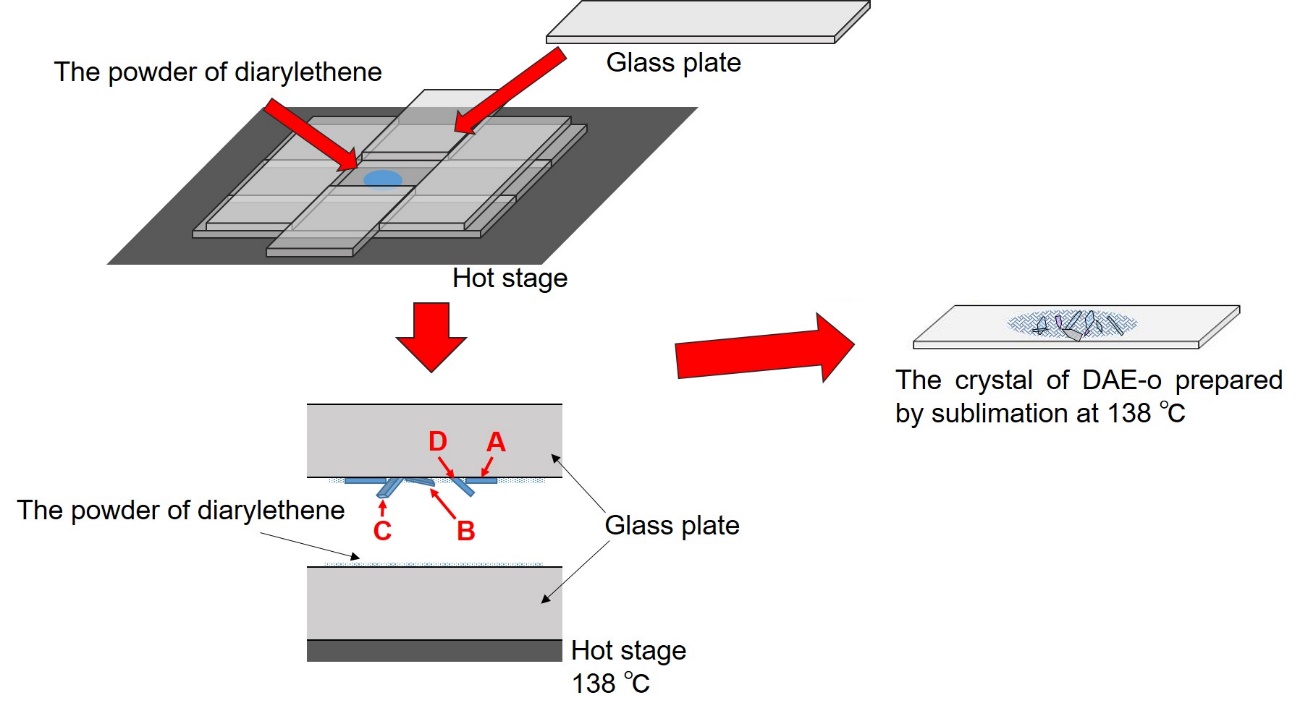
**

**Figure S1.** Formation of crystals of DAE-o and distribution of four different shapes of the crystals. In a 26 × 26 × 1.2 mm space between slide glasses, 1.0 mg of DAE-o was scattered. The glasses were heated on the hotplate for 90 min at 138 °C, which is 20 °C lower than the melting point of DAE-o (158 °C). Four types of crystals of DAE-o were generated on the glass plate on the top: (A) rod-shaped crystals grown along the glass surface, (B) hollow crystals grown along the glass surface, (C) hollow crystals grown standing on the surface, and (D) platelet crystals standing on the surface. Crystals (B), (C), and (D) were grown at the same growing point. In this study, the crystal of (A) was used.

**Table S1.** Crystal data of DAE-o prepared by sublimation

|  | DAE-o | DAE-o UV (after 400 nm　light irradiation for 5 min) |
| --- | --- | --- |
| formula | C_26_H_16_F_8_N_2_S_2_ | C_26_H_16_F_8_N_2_S_2_ |
| formula weight | 572.53 | 572.53 |
| *T* / K | 93(2) | 93(2) |
| crystal system | monoclinic | monoclinic |
| space group | *P*2_1_/*c* | *P*2_1_/*c* |
| *a* / Å | 13.3855(4) | 13.4391(6) |
| *b* / Å | 25.1206(9) | 25.2408(14) |
| *c* / Å | 14.7089(5) | 14.6414(8) |
| *α* / ° | 90 | 90 |
| *β* / ° | 97.3991(10) | 97.8337(16) |
| *γ* / ° | 90 | 90 |
| *V* / Å^3^ | 4904.7(3) | 4920.2(4) |
| *Z* | 8 | 8 |
| *R*_1_ (*I* > 2σ(*I*)) | 0.0318 | 0.0587 |
| *wR*_2_ (*I* > 2σ(*I*)) | 0.0418 | 0.0798 |
| *R*_1_ (all data) | 0.0742 | 0.1423 |
| *wR_2_* (all data) | 0.0793 | 0.1536 |
| CCDC No. | 1453359 | 1476101 |


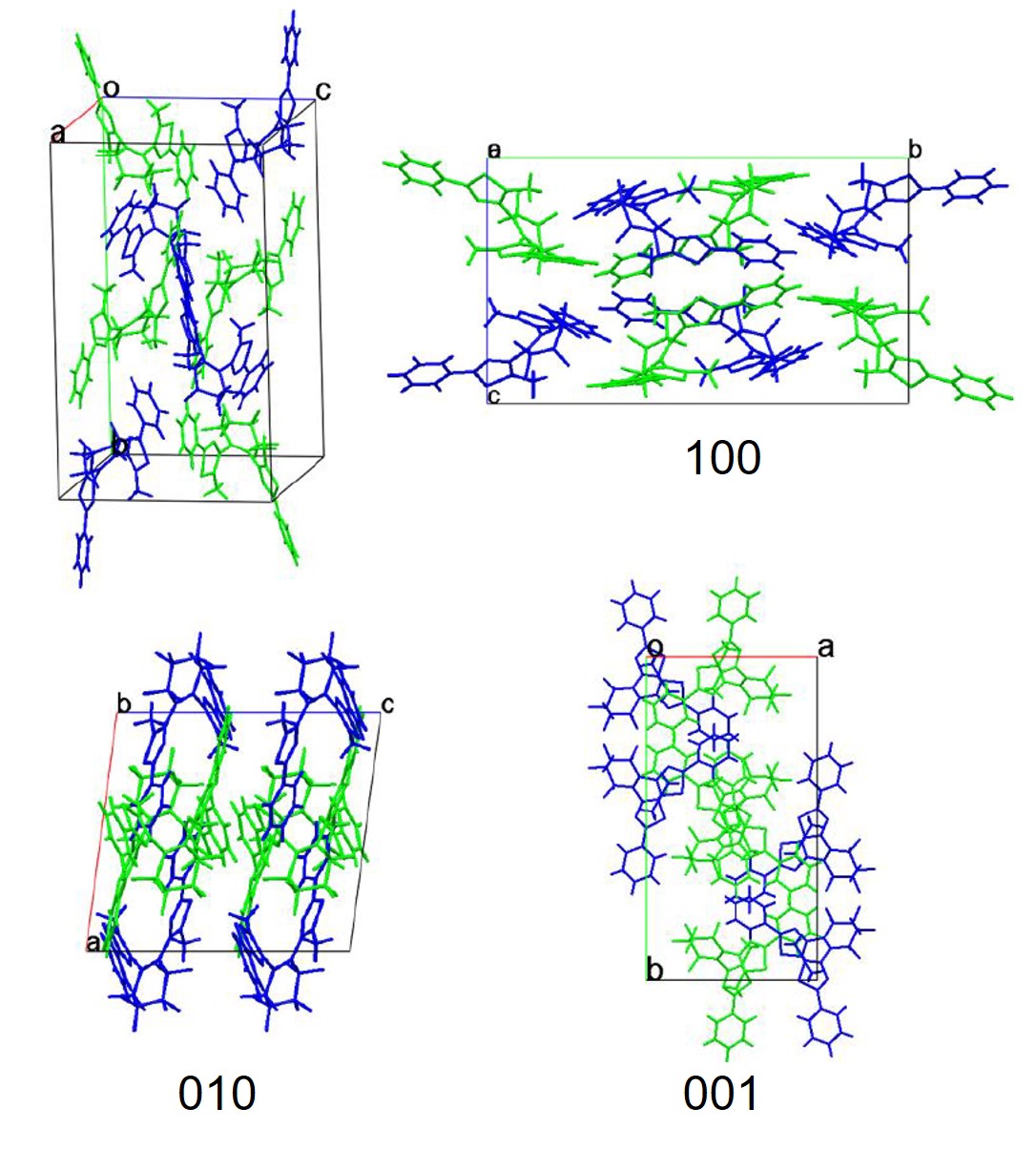


**Figure S2.** Molecular packing in the crystal DAE-o prepared by sublimation.


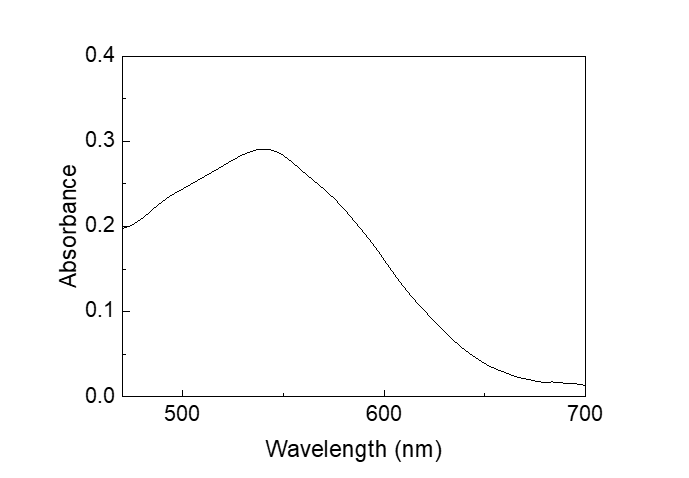


**Figure S3.** Absorption spectrum of DAE in the crystalline state viewed from (010) surface.
